# Correction: Ordered mesoporous zirconium oxophosphate supported tungsten oxide solid acid catalysts: the improved Brønsted acidity for benzylation of anisole

**DOI:** 10.1039/d5ra90033f

**Published:** 2025-04-02

**Authors:** Zhichao Miao, Huahua Zhao, Huanling Song, Lingjun Chou

**Affiliations:** a State Key Laboratory for Oxo Synthesis and Selective Oxidation, Lanzhou Institute of Chemical Physics, Chinese Academy of Sciences Lanzhou 730000 People's Republic of China ljchou@licp.cas.cn +86 931 4968129 +86 931 4968066; b University of Chinese Academy of Sciences Beijing 100049 People's Republic of China; c Suzhou Institute of Nano-Tech and Nano-Bionics, Chinese Academy of Sciences Suzhou 215123 People's Republic of China

## Abstract

Correction for ‘Ordered mesoporous zirconium oxophosphate supported tungsten oxide solid acid catalysts: the improved Brønsted acidity for benzylation of anisole’ by Zhichao Miao *et al.*, *RSC Adv.*, 2014, **4**, 22509–22519, https://doi.org/10.1039/C4RA02809K.

The authors regret an error in Fig. 3 in the original manuscript.

In Fig. 3(2) in the original manuscript, the XRD patterns of sample c (15 wt% WO_3_/M-ZrPO) and d (20 wt% WO_3_/M-ZrPO) were mistakenly duplicated. Fig. 3(2) is a partial (20–30°) magnification of Fig. 3(1), and when Fig. 3(1) was prepared, the data of sample d (20 wt% WO_3_/M-ZrPO) was mistakenly copied twice, resulting in data duplication.

A corrected Fig. 3 is provided below.

**Fig. 3:**
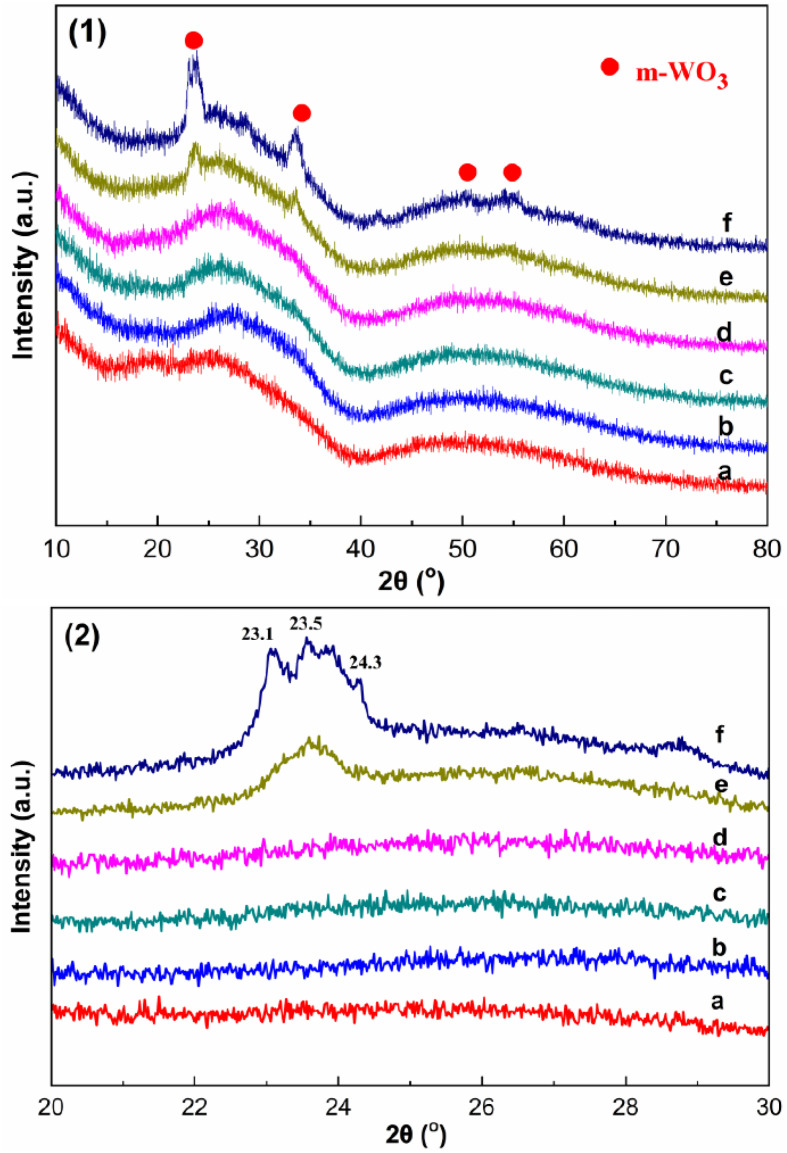
Wide-angle X-ray diffraction patterns of *X* wt% WO_3_/M-ZrPO: (a) 5 wt% WO_3_/M-ZrPO, (b) 10 wt% WO_3_/M-ZrPO, (c) 15 wt% WO_3_/M-ZrPO, (d) 20 wt% WO_3_/M-ZrPO, (e) 25 wt% WO_3_/M-ZrPO, (f) 30 wt% WO_3_/M-ZrPO.

An independent expert has viewed the raw data and corrected figure and has concluded that the data are consistent with the discussions and conclusions presented.

The Royal Society of Chemistry apologises for these errors and any consequent inconvenience to authors and readers.

